# Analysis of the effects of *Bacillus velezensis* HJ-16 inoculation on tobacco leaves based on multi-omics methods

**DOI:** 10.3389/fbioe.2024.1493766

**Published:** 2024-12-06

**Authors:** Qing Zhou, Jinchu Yang, Yingjie Feng, Zongcan Yang, Yixuan Wang, Zhan Zhang, Tingting Zhang, Wenzhao Liu, YongMing Xu, Yongfeng Yang, Jihong Huang

**Affiliations:** ^1^ Technology Center, China Tobacco Henan Industrial Co., Ltd., Zhengzhou, China; ^2^ State Key Laboratory of Crop Stress Adaptation and Improvement, College of Agriculture, Henan University, Kaifeng, China

**Keywords:** tobacco, *Bacillus velezensis*, fermentation, whole-genome sequencing, metagenomic, untargeted metabolomics

## Abstract

In this study, a strain isolated from the surface of flue-cured tobacco leaves, identified as *Bacillus velezensis* HJ-16, was applied in the solid-state fermentation of tobacco leaves. This strain, known for producing thermally stable enzymes, including amylase, cellulase, and protease, significantly improved the sensory qualities of tobacco, enhancing aromatic intensity, density, and softness, while reducing irritation. Whole-genome sequencing and functional annotation revealed that *B. velezensis* HJ-16 possesses a single circular chromosome containing genes associated with enzyme production and metabolic activities, particularly in carbohydrate metabolism and amino acid metabolism. Untargeted metabolomics analysis identified significant changes in non-volatile metabolites induced by fermentation. These metabolites were enriched in pathways related to flavonoid biosynthesis, alkaloid biosynthesis, aromatic amino acid metabolism, lipid metabolism, and carbon metabolism. Metagenomic analysis showed that *Bacillus* became the dominant genus on the tobacco leaf surface following inoculation with *B. velezensis* HJ-16, altering the microbial community composition, reducing diversity and evenness, and enhancing microbial metabolic activity. These findings underscore the potential of *B. velezensis HJ-16* as a biotechnological tool to improve tobacco leaf quality.

## 1 Introduction

Tobacco (*Nicotiana tabacum* L.) is one of the most widely cultivated economic crops worldwide (Mavroeidis et al., 2024). The chemical composition of its leaves plays a crucial role in determining the appearance, quality, and flavor of cigarettes (Hao et al., 2024). Freshly harvested tobacco leaves typically have an imbalanced chemical composition, insufficient aroma, and a high degree of irritation, rendering them unsuitable for cigarette production (Ye et al., 2017). The curing process is essential for facilitating a series of chemical and biochemical transformations within the leaves, enhancing their aroma and reducing irritation. For example, the degradation of carbohydrates during curing contributes to aroma development, while the presence of reducing sugars positively impacts smoking properties by improving both flavor and aroma (Banožić et al., 2020; Gong et al., 2023).

Starch, cellulose, pectin, and protein are fundamental components of tobacco leaves that significantly impact the quality of flue-cured tobacco. High levels of carbohydrates like starch, cellulose, and pectin can lead to unpleasant odors and increase irritation when burned. Similarly, excessive protein content impairs burn performance, increases irritation, and reduces the elasticity and toughness of the leaves. Additionally, proteins serve as precursors to harmful compounds, such as quinoline and hydrogen cyanide (HCN), which compromise tobacco quality and pose safety risks. Therefore, reducing macromolecule content in tobacco leaves is essential for enhancing both quality and usability (Dai et al., 2020; Gong et al., 2023; Jiang et al., 2024). Studies have indicated that microorganisms on the surface of tobacco leaves play a crucial role in degrading these macromolecules during curing. For example, co-fermentation of tobacco leaves with *Bacillus amyloliquefaciens* 44# (with amylase activity) and *Bacillus kochii* 3# (with protease activity) degrades macromolecules into smaller molecules, such as reducing sugars, free amino acids, and short peptides. These degradation products can then participate in Maillard reactions, which increase various aromatic compounds, including 2-pentylfuran, 1-(2-furanmethyl)-1H-pyrrole, furfural, and 2,5-dimethylpyrazine. These compounds, which more than double, contribute to a rich and mellow flavor. Notably, 2-pentylfuran, a key aroma contributor in tobacco, imparts fruity, roasted, and sweet notes (Gong et al., 2023; Wen et al., 2023).

*Bacillus, Pseudomonas*, *Enterobacter*, and *Sphingomonas* are the main dominant genera on flue-cured tobacco leaves (Huang et al., 2010; Zhou et al., 2020), producing extracellular enzymes such as amylase, protease, pectinase, and cellulase, which contribute to the formation of sweet and aromatic flavors (Dai et al., 2020; Gong et al., 2023; Jiang et al., 2024; Ning et al., 2023; Weng et al., 2024). For instance, applying *Bacillus subtilis* BSP1 to tobacco leaves stimulates the production of enzymes essential for protein and cellulose degradation. This application also enhances cell energy production pathways, accelerating protein degradation. Compared to untreated samples, *Bacillus subtilis* BSP1 treated tobacco leaves showed a 36.9% increase in protein degradation rate, with an overall increase in protein degradation of 29% by the end of curing (Jiang et al., 2024). Similarly, *Bacillus subtilis* ZIM3, isolated from cured flue-cured tobacco leaves, can degrade starch and cellulose over a wide temperature and pH range, making it a promising candidate for use in tobacco leaf fermentation (Dai et al., 2020). In addition, some studies have highlighted the potential of using strains that produce both carbohydrate-hydrolyzing enzymes and proteases to significantly reduce curing time and improve tobacco quality (Ma L. et al., 2024; Wen et al., 2023; Wu et al., 2022b; Wu et al., 2021). For example, fermenting tobacco leaves with a microbial suspension containing *Bacillus subtilis* B1 and *Cytobacillus oceanisediminis* C4 effectively degrades protein, starch, and cellulose, accelerating the accumulation of reducing sugars and amino acids, which are important precursors of the Maillard reaction. This process imparts a unique sweetness and caramel aroma to the tobacco (Ning et al., 2023).

The composition and abundance of microbial communities on tobacco leaves are influenced by factors such as geographical location (Zheng et al., 2022a), leaf flavor characteristics (e.g., fresh and strong flavor style flue-cured tobacco leaves) (Zhang et al., 2020), and whether the leaves have been cured (Wu et al., 2022a). Interactions among microbes can significantly affect the metabolic activity of microbial communities (Ghosh et al., 2016; Pierce and Dutton, 2022), thereby influencing the chemical composition of cigar tobacco leaves (Jiang et al., 2024). Introducing appropriate exogenous microorganisms can stimulate microbial interactions, enhance microbial metabolic capacity, and promote tobacco fermentation. For instance, inoculation with *Acinetobacter spp*. on cigar tobacco leaves has been shown to modify existing bacterial communities, with significant correlations to various flavor compounds (Zheng et al., 2022b). Additionally, bacterial communities have a more pronounced effect on cigar tobacco leaf flavor compared to fungal communities (Zheng et al., 2022a). In one study, inoculating *Filobasidium magnum* F7 into flue-cured tobacco leaves and fermenting for 2 days significantly decreased microbial community diversity and richness while increasing the relative abundance of the functional strain F7 from 2% to 64.68%, thereby facilitating effective bio-fermentation (Wu et al., 2022b).

In recent years, metabolomics-based approaches have been increasingly applied to the study of plant leaf fermentation, including tea leaves (Hutasingh et al., 2024; Wu et al., 2024; Ye et al., 2024), mulberry leaves (Wang et al., 2024), turnip leaves (Tomita et al., 2018) and tobacco leaves (Chen et al., 2024; Li et al., 2020; Wu et al., 2023). Metabolites in tobacco leaves are critical determinants of leaf quality (Zhang et al., 2018). Free amino acids from protein hydrolysis and free fatty acids from lipid breakdown are important precursors for flavor substances (Han et al., 2024). Compounds related to the degradation pathways of terpenoids and higher fatty acids can enhance the aroma of tobacco leaves, and are among the key factors in improving the quality of flue-cured tobacco (Wu et al., 2022a). One report found that tobacco leaves harvested after maturation and fermented at 45°C for 6 weeks showed increased concentrations of L-phenylalanine and sphingosine, while the levels of phytosphingosine and nicotine significantly decreased. Pathway analysis revealed that L-phenylalanine is involved in amino acid metabolism, while phytosphingosine and sphingosine participate in sphingolipid metabolic pathways, which are part of lipid metabolism (Li et al., 2020). Additionally, microbes play a crucial role in enhancing cigar tobacco leaf quality by influencing sugar metabolism, amino acid metabolism, and lipid metabolism pathways (Zhang et al., 2023). In summary, the metabolic pathways of terpenoids, amino acids, lipids, and carbon compounds are pivotal during tobacco leaf fermentation, significantly altering metabolite profiles and enhancing the sensory quality of tobacco.

While many functional strains have been screened and applied in tobacco leaf fermentation, several challenges remain. These include extended fermentation times, such as *Bacillus subtilis* ZIM3, which requires 6–8 days for treatment (Dai et al., 2020), or the complexity of co-fermentation with multiple strains (Ning et al., 2023). In this work, we isolated a strain of *Bacillus velezensis* HJ-16 from high-quality flue-cured tobacco leaves, capable of secreting amylase, cellulase, and protease. When applied to solid-state tobacco fermentation, it was able to significantly and efficiently enhance leaf quality in a shorter time. Multi-omics techniques were employed to comprehensively analyze the effects of *B. velezensis* HJ-16 on flue-cured tobacco leaf quality. Whole-genome sequencing was conducted to elucidate the genetic basis for the secretion of various extracellular enzymes by this functional strain. Comparative metabolomics analysis was used to investigate the impact of *B. velezensis* HJ-16 fermentation on the tobacco metabolite profile, identify key metabolites, and elucidate metabolic pathways related to leaf quality and flavor. Additionally, metagenomic sequencing was employed to assess the effects of exogenous microbial inoculation on the structure and function of microbial communities.

## 2 Materials and methods

### 2.1 Detection of extracellular enzymes produced by *Bacillus velezensis* HJ-16

*Bacillus velezensis* HJ-16 was isolated from the surface of flue-cured tobacco leaves harvested in Henan Province in 2022 by the Technology Center of China Tobacco Henan Industrial Co., Ltd. The strain is currently preserved at the China Center for Type Culture Collection (CCTCC) under the accession number M 20231960.

A single colony of *B. velezensis* HJ-16 was grown on a Luria-Bertani (LB) agar plate, then inoculated into LB broth. The culture was incubated at 35°C with shaking at 180 rpm for 48 h. After incubation, the culture was centrifuged at 8,000 × g for 10 min at 4°C to obtain the supernatant containing the extracellular enzymes.

To determine the optimal reaction temperature, the crude enzyme solution was incubated with substrates at various temperatures, followed by enzymatic activity measurement. Protease activity was assessed using the Folin-Ciocalteau method following the Chinese national standard GB/T 23527.1-2023. One unit (U) of protease activity was defined as the amount of enzyme generating 1 μg of tyrosine from casein per minute, measured at pH 7.5 for neutral protease and pH 10.5 for alkaline protease. Alpha-amylase and cellulase activities were quantified using the 3,5-dinitrosalicylic acid (DNS) assay, with one unit defined as the enzyme amount required to produce 1 mg of reducing sugar from soluble starch per hour at pH 5.6 for alpha-amylase, and the amount of enzyme degrading carboxymethyl cellulose sodium to 1 mg of glucose per hour at pH 5.0 for cellulase (Mandels et al., 1962). Enzyme activity was expressed as units per milliliter (U/mL) of crude enzyme solution.

To evaluate the thermal stability of the extracellular enzymes, the enzyme solutions were incubated at different temperatures for 1 or 2 h in water baths, followed by measuring residual enzyme activity at their optimal reaction temperatures.

### 2.2 Preparation of tobacco samples

For preparing the bacterial suspension, *B. velezensis* HJ-16 was inoculated into LB broth, incubated at 35°C with shaking at 180 rpm for 24 h. The culture was centrifuged at 5,000 × g for 10 min at 4°C, the supernatant was discarded, and the bacterial pellet was washed twice with sterile water. The pellet was then resuspended to an optical density (OD_600_) of 2.0 for further use.

Tobacco leaves were obtained from China Tobacco Henan Industrial Co., Ltd. The leaves were cut into tobacco strands and prepared for treatment. The cut tobacco samples were evenly sprayed with either sterile water (control) or a bacterial suspension at a 2% (v/w) inoculation concentration. The experimental groups were designated as Ctrl (water-treated) and HJ_16 (fermented with *B. velezensis* HJ-16). To determine the optimal fermentation conditions, a three-factor, three-level orthogonal experiment was conducted. Fermentation was carried out at 35°C and 70% relative humidity for 72 h, with periodic stirring every 4 h to ensure uniform fermentation. A portion of the tobacco samples was frozen at −80°C for untargeted metabolomics and metagenomic analysis. The remaining samples were subjected to heat treatment at 85°C for 15 min. Sensory evaluation was then performed to assess the impact of *B. velezensis* HJ-16 on enhancing tobacco quality attributes (Wen et al., 2023).

### 2.3 Sensory evaluation and tobacco surface morphology analysis

A panel of seven trained experts conducted the sensory evaluation in accordance with Chinese tobacco industry standards (YC/T 138–1998, YC/T 496-2014) and national standards (GB/T 10221-2021, GB/T 12310-2012) (Li et al., 2020; Wu et al., 2022a; Wu et al., 2022b). A 9-point scale was used to evaluate key sensory attributes, including aromatic quality, intensity, density, softness, aftertaste, foreign odor, and irritation. The final score was calculated using a weighted formula that considers all these characteristics: [(aromatic quality + intensity) × 2.3] + [density × 1.5] + softness + aftertaste + foreign odor + irritation. This formula provides a comprehensive assessment of the tobacco’s sensory properties.

The surface morphology of the water-treated and *B. velezensis* HJ-16 fermented tobacco leaves was examined using scanning electron microscopy (SEM) (Prisma E, Thermo Fisher Scientific, United States). Observations were made under high vacuum with an accelerating voltage of 15.00 kV.

### 2.4 Strain identification

The genomic DNA of *B. velezensis* HJ-16 was extracted and used as a template for polymerase chain reaction (PCR) amplification of the 16S rRNA gene using universal bacterial primers 27F (5′-AGAGTTTGATCMTGGCTCAG-3′) and 1492R (5′-GGT​TAC​CTT​GTT​ACG​ACT​T-3′). The amplified 16S rRNA sequences were then compared for similarity using BLAST in the NCBI database. The evolutionary tree was inferred using the Neighbor-Joining method (Saitou and Nei, 1987) with 1,000 replicates in MEGA 11 software. Evolutionary distances were calculated using the p-distance method and all positions with less than 50% site coverage were eliminated (Tamura et al., 2021).

### 2.5 Whole-genome sequencing analysis

The genomic DNA of *B. velezensis* HJ-16 was extracted following the STE method, detected by agarose gel electrophoresis, and quantified with Qubit. The genome was sequenced using Single Molecule, Real-Time (SMRT) technology at Beijing Novogene Bioinformatics Technology Co., Ltd. Low-quality reads were filtered using SMRT Link v8.0, and the remaining high-quality reads were assembled with Canu software to produce a single, gap-free contig.

Gene function annotation was performed using NR (Non-Redundant Protein Database), GO (Gene Ontology), COG (Clusters of Orthologous Groups), KEGG (Kyoto Encyclopedia of Genes and Genomes), and CAZy (Carbohydrate-Active enZYmes) databases. A whole-genome BLAST search (E-value < 1e-5, minimal alignment length percentage > 40%) was conducted against these databases to identify gene functions.

### 2.6 Untargeted metabolomics analysis

Fermented tobacco samples (100 mg) were ground with liquid nitrogen, resuspended in prechilled 80% methanol, and followed by vigorous vortexing. Subsequently, the samples were incubated on ice for 5 min and then centrifuged at 15,000 × g at 4°C for 20 min. A portion of the supernatant was diluted with LC-MS grade water to a final concentration of 53% methanol, transferred to a fresh tube, and centrifuged again at 15,000 × g at 4°C for 20 min. The final supernatant was injected into the LC-MS/MS system for analysis (Want et al., 2013).

Samples were injected into a Hypersil Gold column (100 × 2.1 mm, 1.9 μm) using a 12-min linear gradient at a flow rate of 0.2 mL/min. The eluents for positive and negative polarity modes were eluent A (0.1% formic acid in water) and eluent B (methanol). The solvent gradient was programmed as follows: 2% B for 1.5 min; 2%–85% B for 3 min; 85%–100% B for 10 min; 100%–2% B for 10.1 min; 2% B for 12 min.

The Q Exactive HF mass spectrometer operated in both polarity mode with a spray voltage of 3.5 kV, capillary temperature of 320°C, sheath gas flow rate of 35 psi, auxiliary gas flow rate of 10 L/min, S-lens RF level of 60, and auxiliary gas heater temperature of 350°C. The UHPLC-MS/MS analyses were conducted using a Vanquish UHPLC system coupled with an Orbitrap Q Exactive HF mass spectrometer at Novogene Co., Ltd. (Beijing, China).

Metabolites were annotated using the KEGG and LIPIDMaps databases. Partial Least Squares Discriminant Analysis (PLS-DA) was conducted using metaX software (Wen et al., 2017). Statistical significance was determined through univariate analysis (t-test) with *p* < 0.05. Metabolites with p < 0.05, Variable Importance in Projection (VIP) scores > 1, and fold changes (Fc) > 1.2 or < 0.83 were classified as differential metabolites.

Volcano plots were generated based on log2(Fc) and -log10 (p-value) using ggplot2 in R. Clustering heat maps were created using z-scores of differential metabolites’ intensity areas and plotted with the Pheatmap package in R. Metabolite functions and pathways were analyzed using the KEGG database, with pathway enrichment considered statistically significant when the ratio x/n > y/N and p < 0.05. Here, N represents the total number of metabolites annotated to all KEGG pathways, n is the number of differential metabolites among N, y is the number of metabolites annotated to a specific KEGG pathway, and x is the number of differential metabolites enriched in that pathway. Pathway enrichment analysis was conducted following the methods reported by Tang et al. (Tang et al., 2023).

### 2.7 Metagenomic sequencing analysis

Genomic DNA was extracted from the tobacco samples and its quality was assessed through agarose gel electrophoresis and AATI peak detection. A 1 μg sample of genomic DNA was then used for library construction. Afterward, the integrity and fragment size of the library were evaluated with AATI analysis. Libraries that passed quality control were pooled according to their effective concentrations and target data output requirements, followed by sequencing using the PE150 platform at Novogene Co., Ltd. (Beijing, China).

Raw data from the Illumina sequencing platform were preprocessed using Fastp (https://github.com/OpenGene/fastp) to obtain clean data for further analysis. The clean data were then assembled using MEGAHIT software, with scaffolds containing N removed. Bowtie2 software was used to filter out reads originating from the host tobacco. Additionally, Solanaceae sequences were filtered based on annotations in the NR database.

Gene prediction for scaffolds (≥ 500 bp) in each sample was performed using MetaGeneMark (http://topaz.gatech.edu/GeneMark/). Clean data from each sample were aligned to the initial gene catalogue using Bowtie2 to determine the read counts for each gene. Gene abundance was calculated based on read counts and gene length. Statistical analyses included basic statistical summaries, α- and β-diversity analyses, and Linear Discriminant Analysis Effect Size (LEfSe). Species and functional annotations were performed using DIAMOND software.

### 2.8 Statistical analysis

Each experimental group included at least three biological replicates. Data were presented as mean ± standard error of the mean (SEM). Statistical significance was evaluated using Student’s unpaired t-test and one-way ANOVA with Tukey’s post-hoc test, with significance set at *p* < 0.05.

## 3 Results

### 3.1 Characterization of extracellular enzymes produced by the HJ-16 strain

**FIGURE 1:**
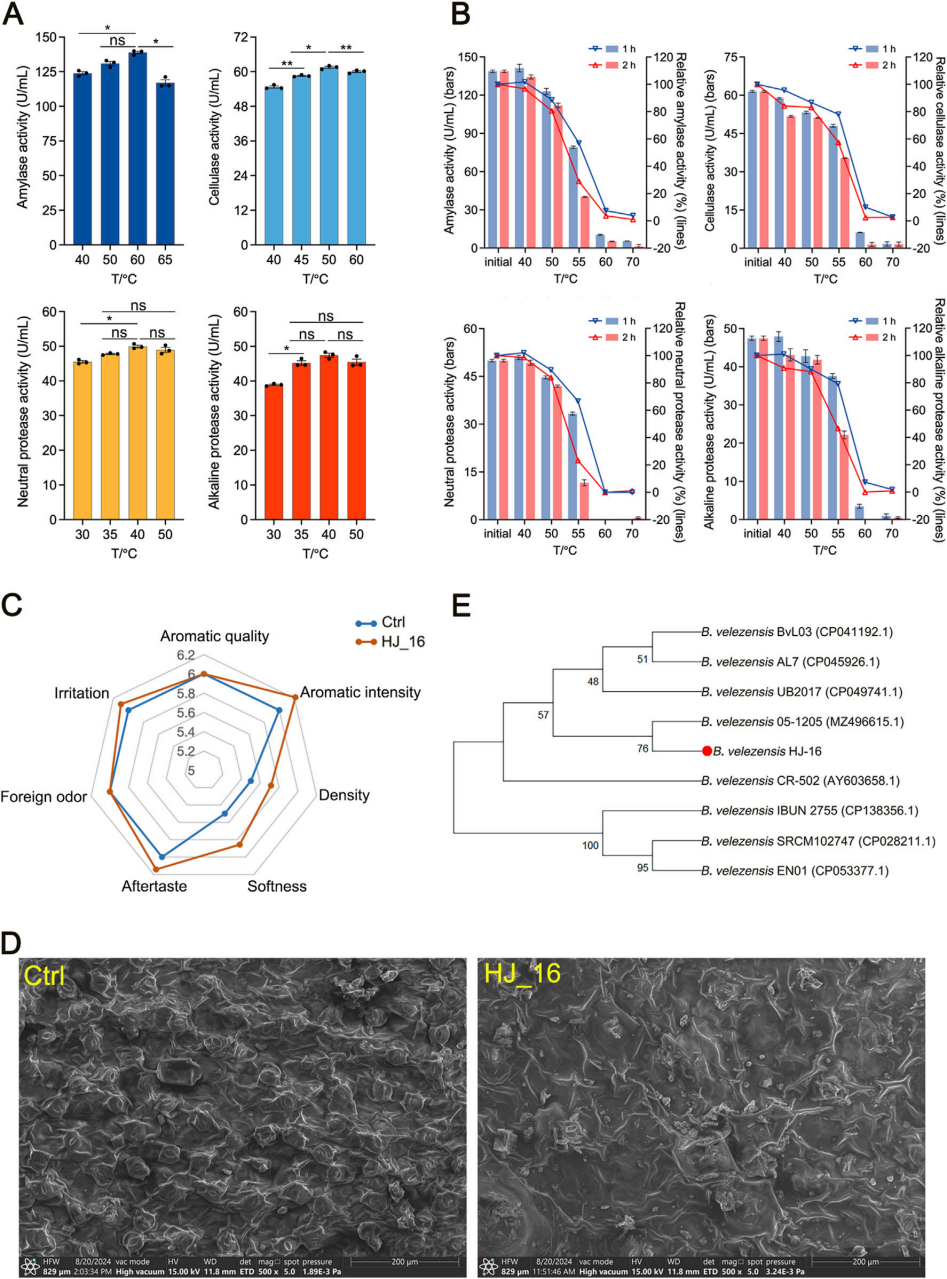
Enzymatic properties, strain identification, and fermentation tobacco leaves by the HJ-16 strain. **(A)** Enzyme activity of the HJ-16 strain at different temperatures. Statistical analysis was performed using one-way ANOVA with Tukey’s post-hoc test. Significance is indicated as *p < 0.05, **p < 0.01, and ns indicating not significant (*p* > 0.05). **(B)** The enzyme activity and relative enzyme activity of the HJ-16 strain after 1 and 2 h of incubation in water baths at different temperatures. **(C)** Sensory evaluation radar chart of tobacco leaves fermented by the HJ-16 strain. **(D)** Scanning electron microscopy images showing the comparison between water-treated and HJ-16-treated tobacco leaf surface. **(E)** Phylogenetic tree of the HJ-16 strain based on 16S rRNA sequences, constructed using the Neighbor-Joining method. Branches show the percentage of replicate trees (1,000 replicates). Evolutionary analysis was performed using MEGA11.

The HJ-16 strain produces several extracellular enzymes, including amylase, cellulase, neutral protease, and alkaline protease. Optimal temperature ranges for these enzymes were determined through quantitative assays. Amylase and cellulase exhibited peak activities at 50°C–60°C and 50°C, respectively. Neutral protease and alkaline protease showed their highest activities between 35°C–50°C. The highest enzymatic activities produced by the HJ-16 strain were measured as follows: amylase at 138.8 U/mL, cellulase at 61.5 U/mL, neutral protease at 49.9 U/mL, and alkaline protease at 47.4 U/mL (Figure 1A).

To evaluate thermal stability, the enzymes were incubated at various temperatures for 1 or 2 h in water baths, followed by activity measurements. After 1-h incubation at 55°C, amylase and neutral protease retained approximately 60% of their initial activity, while cellulase and alkaline protease maintained around 80% (Figure 1B). The results suggest that the enzymes produced by the HJ-16 strain are suitable for industrial bioprocessing. Collectively, these enzymes endow the HJ-16 strain with the potential to degrade complex biomacromolecules in tobacco leaves, such as starch, cellulose, and proteins.

### 3.2 Sensory evaluation and SEM analysis

The results from a three-factor, three-level orthogonal experiment indicate that the optimal conditions for solid-state fermentation of tobacco leaves using the HJ-16 strain were the fermentation time of 72 h, fermentation temperature of 35°C, and 2% (v/w) inoculum amount (Table 1). Sensory evaluation radar chart showing the flavor profile of tobacco leaves. Tobacco leaves fermented with the HJ-16 strain showed a significant improvement in sensory qualities compared to the control group. The overall sensory evaluation score increased by 1.4 points, reaching a total score of 60.8. This improvement includes heightened aromatic intensity, density, and improved softness, as well as reduced irritation, thereby elevating the overall quality of lower-grade tobacco leaves (Figure 1C).

**TABLE 1:** Orthogonal experimental design and results.

(A) Factor levels used in orthogonal experiments			
Factor	A: Fermentation time (h)	B: Fermentation temperature (°C)	C: Inoculum amount (%)
1	48	30	2
2	72	35	3
3	96	40	4

(B) Treatment combinations and sensory evaluation scores				
Treatment	A: Fermentation time (h)	B: Fermentation temperature (°C)	C: Inoculum amount (%)	Sensory evaluation scores
T1	1 (48)	1 (30)	1 (2)	59.32
T2	1 (48)	2 (35)	3 (4)	60.07
T3	1 (48)	3 (40)	2 (3)	57.45
T4	2 (72)	1 (30)	3 (4)	58.93
T5	2 (72)	2 (35)	2 (3)	60.55
T6	2 (72)	3 (40)	1 (2)	59.42
T7	3 (96)	1 (30)	1 (2)	58.89
T8	3 (96)	2 (35)	1 (2)	59.43
T9	3 (96)	3 (40)	3 (4)	58.02

(C) Factor-level averages					
Factor	Level 1	Level 2	Level 3	R (range)	Optimal level
1	58.95	59.63	58.78	0.85	A2
2	59.05	60.02	58.30	1.72	B2
3	59.27	59.00	59.01	0.27	C1

Scanning electron microscopy (SEM) was employed to compare the surface structure of control tobacco leaves and those fermented with the HJ-16 strain (Figure 1D). The control sample (Figure 1D, left) displayed distinct, densely packed porous structures on the leaf surface. In contrast, the sample fermented with the HJ-16 strain (Figure 1D, right) exhibited fewer pores, a looser structure, and some ruptured epidermal cells with particle-like material released. These results suggest that microbial fermentation significantly alters the surface structure of tobacco leaves.

### 3.3 Strain identification

The HJ-16 strain was identified through 16S rRNA gene sequencing. The sequence was analyzed using BLAST, and a phylogenetic tree was constructed via the Neighbor-Joining method in MEGA 11. This analysis confirmed that the HJ-16 strain is closely related to *Bacillus velezensis*, identifying it as *B. velezensis* HJ-16 (Figure 1E).

### 3.4 Whole-genome sequencing analysis

#### 3.4.1 Genomic analysis

**FIGURE 2:**
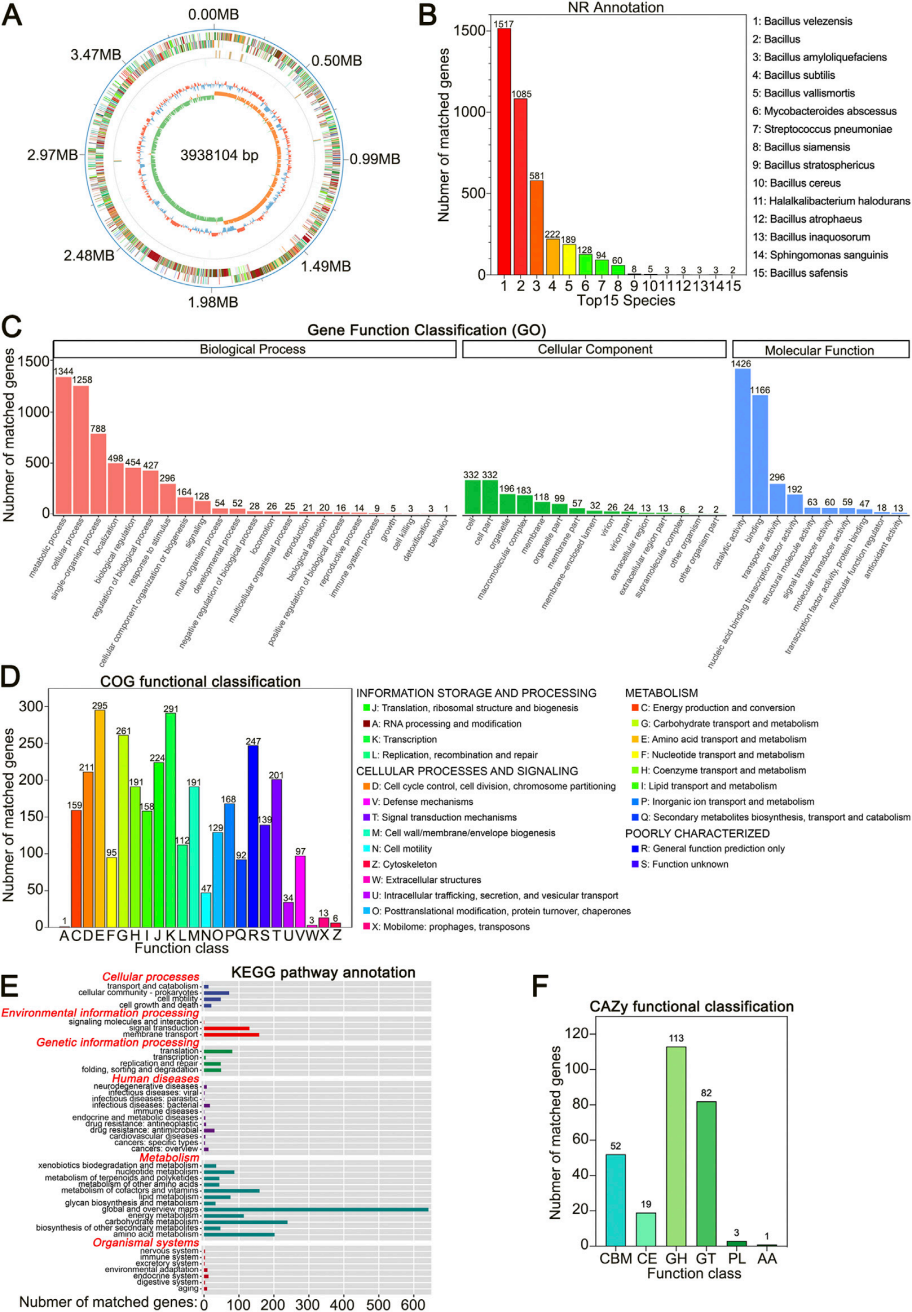
Whole-Genome sequencing of *Bacillus velezensis* HJ-16. **(A)** Circular genome map of *Bacillus velezensis* HJ-16, with genomic coordinates shown in the outermost ring. The concentric rings show, from outer to inner, COG annotations, non-coding RNA (ncRNA), GC content (blue indicating below-average and red indicating above-average values), and GC skew (green for lower G content and orange for higher). **(B)** NR database annotation showing the top 15 species and their respective gene counts. **(C)** GO database functional classification. **(D)** COG database functional classification. **(E)** KEGG pathway annotation. **(F)** CAZy database functional classification.

Whole-genome sequencing of *B. velezensis* HJ-16 using PacBio technology revealed a single circular chromosome consisting of 3,938,104 base pairs (bp) with a GC content of 46.5%. The genome contains 4,012 predicted coding sequences, covering 90.04% of its total length (Figure 2A; Table 2). This genomic information provides insights into the genetic basis of *B. velezensis* HJ-16 to degrade tobacco leaf components, such as starch, cellulose, and proteins, primarily through the analysis of carbohydrate metabolism enzyme genes and related metabolic pathways. Functional gene annotation using the Non-Redundant Protein (NR) database identified 1,517 genes associated with *B. velezensis*, aligning with the results from the 16S rRNA phylogenetic analysis (Figure 2B). The genomic sequence has been deposited in the National Center for Biotechnology Information (NCBI) database (Accession Number: CP169535).

**TABLE 2:** Genome component analysis.

Characteristics	Value
Total genome length (bp)	3,938,104
GC content in genome sequence (%)	46.5
Total number of annotated genes	4,012
Total length of annotated genes (bp)	3,545,727
Proportion of genome occupied by genes (%)	90.04
GC content in gene sequences (%)	47.22
Average gene length (bp)	884

#### 3.4.2 GO and COG database annotations

Gene Ontology (GO) database annotation identified 2,556 genes in *B. velezensis* HJ-16, with 48 GO terms primarily involved in biological processes (BP) and molecular functions (MF), while fewer genes were associated with cellular components (CC). Metabolic processes and cellular processes were the most prominent in the BP domain, with 1,344 and 1,258 genes, respectively, indicating high metabolic activity in *B. velezensis* HJ-16. In the MF domain, catalytic activity and binding were the prominent functional categories, suggesting the strain’s high catalytic potential and substrate-binding capabilities (Figure 2C; Supplementary Table S1).

The Cluster of Orthologous Groups (COG) database annotated 2,995 genes (Figure 2D; Supplementary Table S2), of which 1,419 genes were related to metabolism. This included 261 genes associated with carbohydrate transport and metabolism and 295 genes linked to amino acid transport and metabolism, respectively. These findings provide further molecular evidence of the strain’s ability to degrade carbohydrates and proteins.

#### 3.4.3 KEGG database annotation

Annotation through the Kyoto Encyclopedia of Genes and Genomes (KEGG) database identified 3,853 genes in *B. velezensis* HJ-16, with a significant number of genes linked to metabolic pathways (Figure 2E; Supplementary Table S3). Notably, beyond global metabolism, the largest categories were carbohydrate metabolism and amino acid metabolism, with 239 and 202 genes, respectively. This extensive metabolic capacity supports the strain’s ability to degrade complex biomacromolecules in tobacco leaves. Furthermore, there was notable gene annotation in cofactors and vitamins metabolism, membrane transport and signal transduction pathways, which are critical for nutrient uptake and cellular communication.

#### 3.4.4 CAZy database annotation

The Carbohydrate-Active enZYmes (CAZy) database provides detailed information on enzyme families involved in carbohydrate degradation, modification, and biosynthesis (Cantarel et al., 2009). Using this database, 270 genes associated with carbohydrate-active enzymes were identified in *B. velezensis* HJ-16. Of these, glycoside hydrolases (GHs) constituted the largest proportion at 41.9%, followed by glycosyltransferases (GTs) at 30.4%, and carbohydrate-binding modules (CBMs) at 19.3% (Figure 2F; Supplementary Table S4).

The genome encodes 22 genes in the GH13 family for alpha-amylase (EC 3.2.1.1) and alpha-glucosidase (EC 3.2.1.20). Additionally, the GH57 family includes two genes for alpha-amylase, and the GH4 family contains five genes for alpha-glucosidase. CBMs from the CBM26 and CBM34 families likely enhance the binding of starch-degrading enzymes to their substrates, facilitating the starch degradation process.

Seventeen genes encoding β-glucosidase (EC 3.2.1.21) were identified in the GH1, GH3, and GH5 families. Five genes encoding endoglucanase (EC 3.2.1.4) and cellobiohydrolase (EC 3.2.1.91) were found in the GH5, GH9, and GH51 families. These cellulose-degrading genes likely interact with CBMs from the CBM3, CBM6, and CBM8 families, enhancing cellulose degradation. In summary, the presence of these genes at the genomic level suggests that *B. velezensis* HJ-16 has the potential to effectively degrade both starch and cellulose.

### 3.5 Untargeted metabolomics analysis

#### 3.5.1 Metabolic profiling of tobacco leaves

Previous studies have demonstrated that microbial fermentation can significantly alter metabolite profiles. To assess the biochemical composition changes in tobacco leaves following fermentation with *B. velezensis* HJ-16, untargeted metabolomic analysis using liquid chromatography-mass spectrometry (LC-MS) was performed. A total of 1,405 metabolites were identified and categorized into several categories: lipids and lipid-like molecules (438 compounds), organoheterocyclic compounds (156), phenylpropanoids and polyketides (118), organic acids and derivatives (111), benzenoids (102), organic oxygen compounds (78), alkaloids and derivatives (31), nucleosides, nucleotides, and analogues (28), organic nitrogen compounds (16), lignans, neolignans and related compounds (12), carbohydrates and carbohydrate conjugates (5), and mixed metal/non-metal compounds (1) (Supplementary Table S5).

**FIGURE 3:**
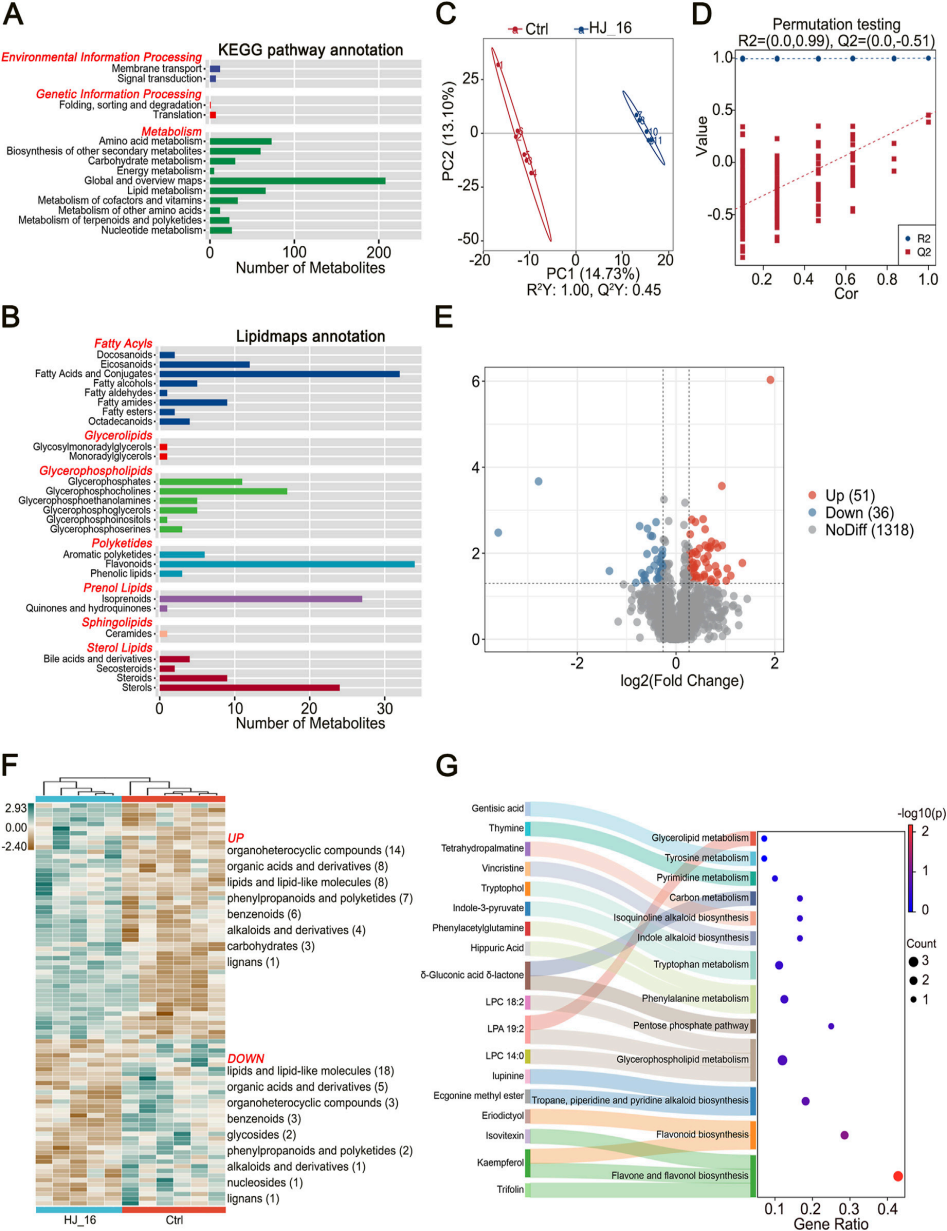
Untargeted metabolomics analysis by LC-MS. **(A)** KEGG pathway annotation for all detected metabolites. **(B)** Lipidmaps annotation based on all detected metabolites. **(C)** PLS-DA model plot. **(D)** PLS-DA permutation test plot. **(E)** Volcano plot displaying differential metabolites (DMs) identified based on *p* < 0.05, VIP score > 1, and fold change (Fc) > 1.2 or < 0.83. **(F)** Heatmap clustering of differential metabolites. **(G)** KEGG pathway enrichment map.

KEGG pathway enrichment analysis indicated that these metabolites were primarily involved in amino acid metabolism (73 compounds), lipid metabolism (66), biosynthesis of other secondary metabolites (60), cofactors and vitamins metabolism (33), carbohydrate metabolism (30), nucleotide metabolism (26), and terpenoids and polyketides metabolism (23) (Figure 3A). Further analysis with LipidMaps revealed a higher abundance of non-volatile lipid metabolites, such as flavonoids (34 compounds), fatty acids and conjugates (32), isoprenoids (27), and sterols (24), which are important contributors to the flavor profile of tobacco leaves (Figure 3B).

#### 3.5.2 Profiles of significant differential metabolites

To identify key biomarkers and distinguish between the control and treated samples, partial least squares discriminant analysis (PLS-DA), a supervised statistical method was applied. This analysis revealed distinct clustering between the Ctrl and HJ_16 groups (Figure 3C). The PLS-DA model showed a high R^2^Y value of 1.00, indicating an excellent fit, while the Q^2^Y value of 0.45 demonstrated moderate predictive ability. No overfitting was observed based on 200 permutation tests (Q^2^ = −0.51), confirming the model’s reliability and accuracy in classifying samples and screening biomarkers (Figure 3D).

The variable importance in projection (VIP) scores quantified the contribution of each compound to the classification. Using criteria of *p* < 0.05, VIP score >1, and fold change (Fc) > 1.2 or < 0.83, a total of 87 significant differential metabolites (DMs) were identified, with 51 upregulated and 36 downregulated (Figure 3E; Supplementary Table S6). Hierarchical cluster analysis (HCA) further grouped the water and HJ-16 fermentation-treated samples into distinct clusters, confirming the significant metabolic changes induced by *B. velezensis* HJ-16 fermentation. The upregulated DMs, visualized on the heatmap, included organoheterocyclic compounds (14), organic acids and derivatives (8), lipids and lipid-like molecules (8), phenylpropanoids and polyketides (7), benzenoids (6), alkaloids and derivatives (4), carbohydrates (3), and lignans (1). Downregulated metabolites were lipids and lipid-like molecules (18), followed by organic acids and derivatives (5), organoheterocyclic compounds (3), benzenoids (3), glycosides (2), phenylpropanoids and polyketides (2), alkaloids and derivatives (1), nucleosides (1), and lignans (1) (Figure 3F). These results provide valuable insights into the metabolic regulation during fermentation.

#### 3.5.3 KEGG pathway enrichment analysis

To further investigate the metabolic pathway alterations induced by *B. velezensis* HJ-16 fermentation, KEGG pathway enrichment analysis was performed. Out of 296 metabolites mapped to KEGG pathways, 18 significant differential metabolites were enriched in 13 distinct metabolic pathways (Figure 3G). The degree of enrichment was represented by the Gene Ratio on the horizontal axis, with larger bubbles indicating more differential metabolites in the pathway.

Important enriched pathways included flavone and flavonol biosynthesis, the flavonoid biosynthesis pathway, as well as aromatic amino acid pathways. Aromatic amino acids, such as phenylalanine, tyrosine, and tryptophan, could be metabolized into phenolic and indolic compounds, contributing to the enhanced flavor and aroma of the tobacco leaves. Additionally, the fermentation process enriched multiple alkaloid metabolism pathways, including tropane, piperidine, pyridine, indole, and isoquinoline alkaloid biosynthesis. Other enriched pathways included glycerophospholipid and glycerolipid metabolism, carbon metabolism, and the pentose phosphate pathway, all contributing to improved sensory characteristics in the fermented tobacco leaves. These results highlight the substantial impact of *B. velezensis* HJ-16 fermentation on the flavonoid, amino acid, alkaloid, lipid, and carbohydrate profiles of tobacco leaves.

### 3.6 Metagenomic sequencing analysis

#### 3.6.1 Diversity analysis

**FIGURE 4:**
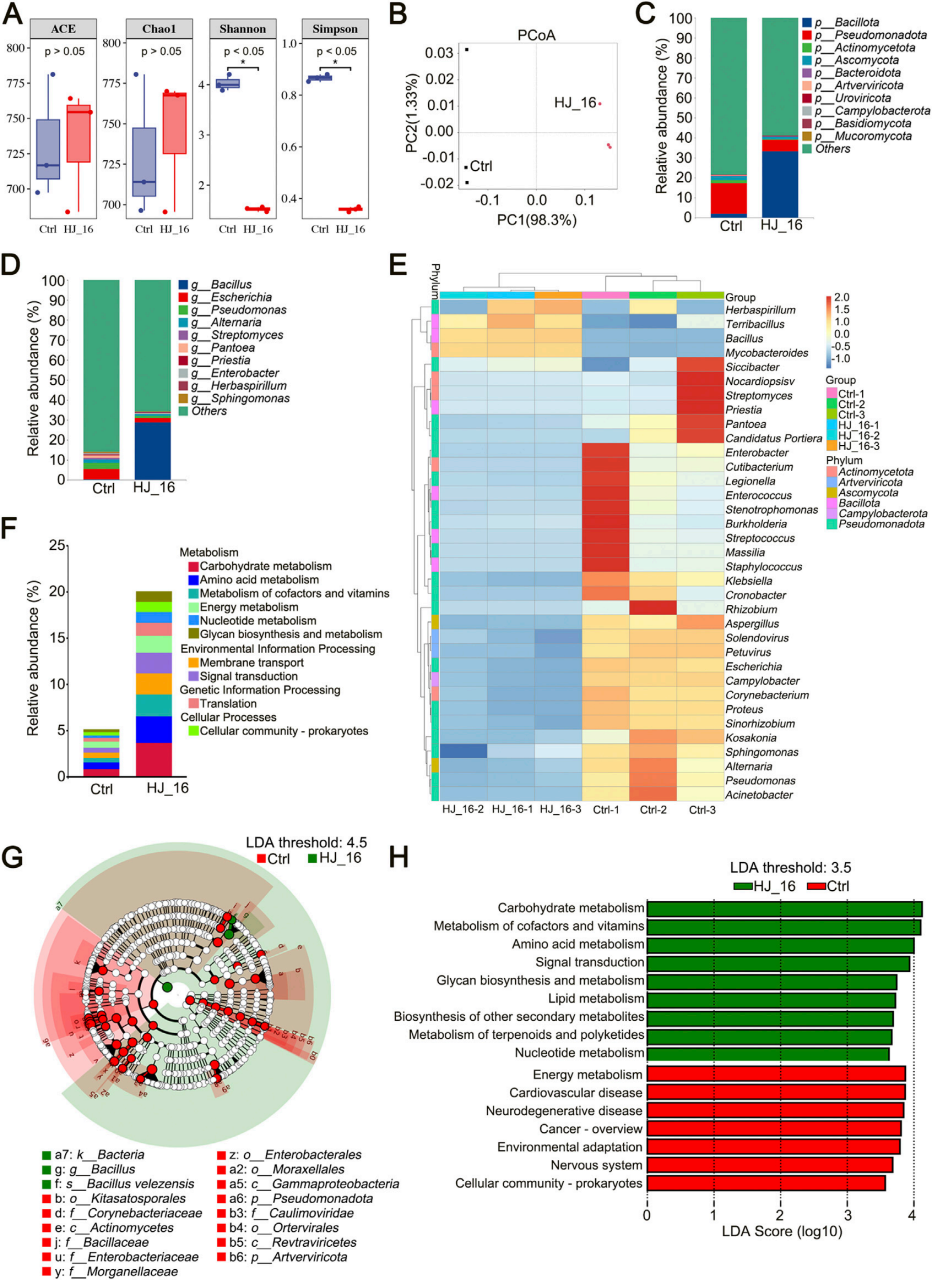
Metagenomic analysis of microbial communities on tobacco leaf surface. **(A)** Alpha diversity analysis. **(B)** Principal Coordinate Analysis (PCoA) based on Bray-Curtis distances. **(C)** Relative abundance of microbial communities at the phylum level. **(D)** Relative abundance of microbial communities at the genus level. **(E)** Heatmap showing the top 35 genera based on relative abundance. **(F)** Relative abundance of KEGG pathways at level 2. **(G)** Cladogram showing biomarkers identified using LDA with a threshold score of 4.5. From outer to inner, the rings represent kingdom, phylum, class, order, family, genus, and species levels. For the control group, only biomarkers from the phylum, class, order, and family levels are displayed. **(H)** LDA of significantly different metabolic pathways with a threshold score of 3.5.

The α-diversity indices, ACE and Chao1, indicated no significant changes in species richness following inoculation with *B. velezensis* HJ-16. However, there was a marked decrease in species evenness, as shown by the Shannon and Simpson indices (*p* < 0.05) (Figure 4A). This suggests that although the total number of species remained relatively stable, their distribution became more uneven, with certain species becoming dominant (Avolio et al., 2019). Principal Coordinate Analysis (PCoA), based on Bray-Curtis distances, revealed significant structural differences in microbial communities between the Ctrl and HJ_16 groups. The first principal coordinate (PC1) explained 98.3% of the total variation, indicating that a few key species were primarily responsible for the observed differences in community composition (Figure 4B). These results indicate that *B. velezensis* HJ-16 inoculation did not significantly affect species richness but had a notable impact on both α- and β-diversity, leading to distinct changes in microbial community structure. This finding is consistent with previous research, which also observed a reduction in Shannon diversity after microbial inoculation (Song et al., 2024).

#### 3.6.2 Relative abundance of microbial communities

Microbial inoculants play a role in modulating microbial community succession. Relative abundance analysis showed that bacteria were the dominant microorganisms on the tobacco leaf surface. The bacterial abundance increased from 20.43% in the Ctrl group to 66.02% in the HJ_16 group, while fungal abundance decreased from 2.34% to 1.32% (Table 3). To further investigate the effect of *B. velezensis* HJ-16 inoculation, the top 10 phyla and genera of relative abundance were analyzed (Figures 4C, D). The dominant phyla included *Bacillota*, *Pseudomonadota*, *Actinomycetota*, *Ascomycota*, and *Bacteroidota*, while the main genera were *Bacillus, Escherichia*, *Pseudomonas*, *Alternaria*, *Streptomyces* and *Pantoea*. Post-inoculation, significant shifts were observed at the phylum level, with the abundance of *Pseudomonadota* decreasing from 15.17% to 5.88%, while *Bacillota* increasing from 1.89% to 33.09%. At the genus level, *Escherichia* dropped from 5.21% to 2.21% and *Pseudomonas* from 2.94% to 1.05%, while *Bacillus* significantly increased from 0.23% to 28.68%, becoming the dominant genus. Using the MetaGenomeSeq method, *Bacteria*, *Bacillota* and *Bacillus* were identified as having significant differences between the two groups (*p* < 0.01) (Supplementary Figure S1). A heatmap of the top 35 genera highlighted the distinct microbial communities present in both groups at the genus level (Figure 4E).

**TABLE 3:** Relative abundance of microbial communities at kingdom level.

Kingdom	Ctrl group relative abundance (%)	HJ_16 group relative abundance (%)
*k__Bacteria*	20.43	66.02
*k__Eukaryota*	2.34	1.32
*k__Viruses*	0.35	0.16
*k__Archaea*	0.02	0.00
others	76.86	32.50

#### 3.6.3 KEGG database annotation and LEfSe analysis

KEGG database analysis was conducted to predict the metabolic functions of the microbial community based on relative abundance data. Among the top 10 KEGG pathways at level 2, significant functional differences were observed between the groups. The HJ_16 group showed increased relative abundances in pathways related to carbohydrate metabolism, amino acid metabolism, and the metabolism of cofactors and vitamins, while the Ctrl group exhibited more uniform functional distributions. These findings suggest that *B. velezensis* HJ-16 inoculation promotes the development of key metabolic modules, leading to substantial changes in the functional structure of the microbial community (Figure 4F).

Linear Discriminant Analysis Effect Size (LEfSe) analysis identified key biomarkers with an LDA score threshold of 4.5, revealing that the Ctrl group had 40 biomarkers compared to only 3 in the HJ_16 group. The cladogram displayed significant biomarkers across phylum, class, order, and family levels for the Ctrl group, while the HJ_16 group showed all distinct biomarkers (Figure 4G).

Additionally, LEfSe was used to identify significant differences in KEGG functional categories between the two groups. Using an LDA score threshold of 3.5, the Ctrl group showed enrichment in seven pathways related to energy metabolism, human diseases, environmental adaptation, and cellular processes. In contrast, the HJ_16 group was enriched in various metabolic pathways, including carbohydrate metabolism, cofactor and vitamins metabolism, amino acid metabolism, glycan biosynthesis and metabolism, lipid metabolism, biosynthesis of other secondary metabolites, terpenoid and polyketide metabolism, and nucleotide metabolism. In addition, signal transduction pathway was identified as biomarker in the HJ_16 group (Figure 4H). These findings support previous studies demonstrating that functional strain inoculation enhances microbial metabolism and facilitates tobacco leaf fermentation (Song et al., 2024; Wu et al., 2022b).

#### 3.6.4 Correlation analysis between metabolites and key microbial genera

**FIGURE 5:**
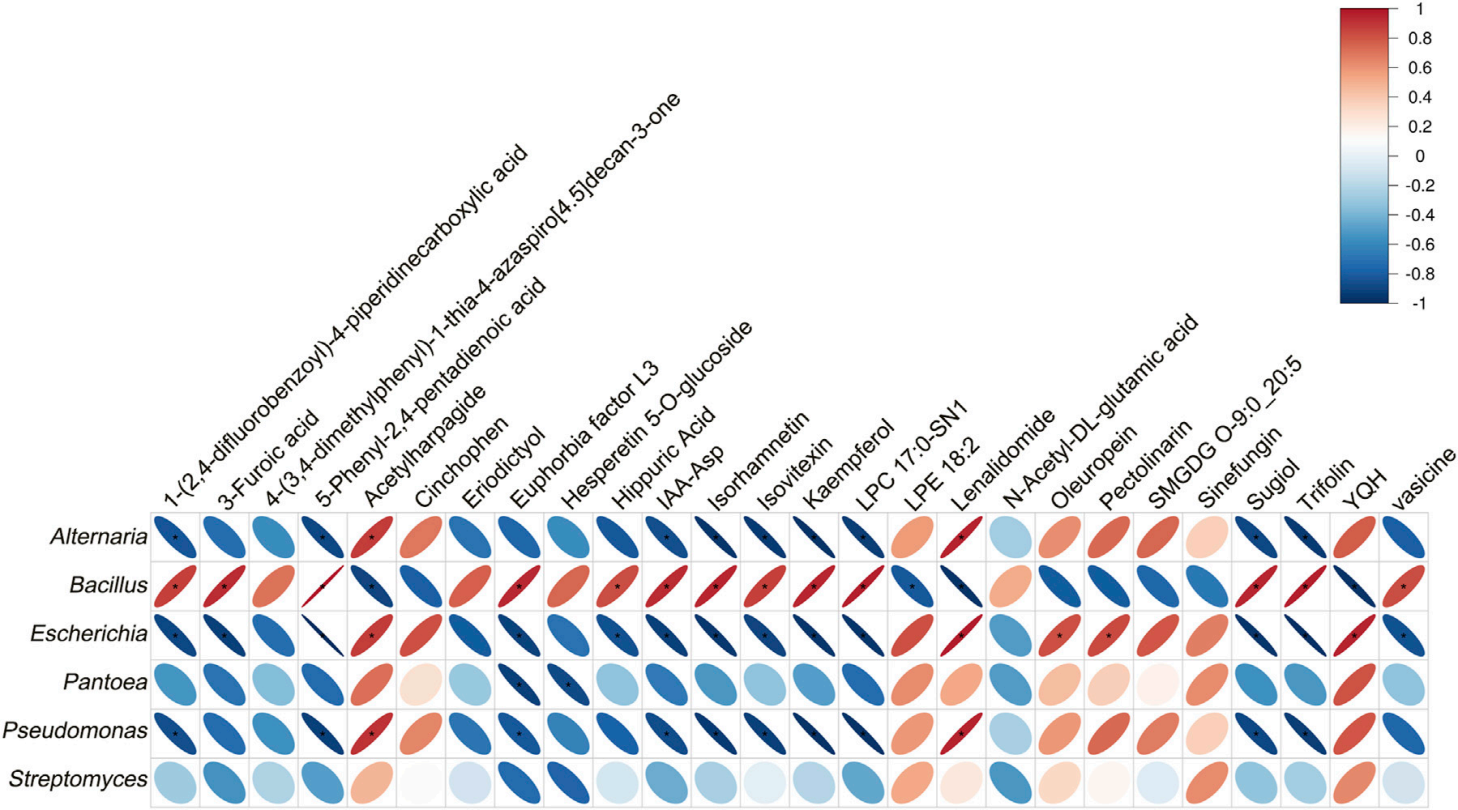
Pearson correlation analysis between differential metabolites and key microbial genera. Microbial genera are displayed along the vertical axis, while differential metabolites are on the horizontal axis. The legend indicates the correlation coefficient, with red representing positive correlations and blue representing negative correlations. Significance is marked as *p* < 0.05.

From the untargeted metabolomics data, 26 differential metabolites (DMs) were identified based on the criteria of *p* < 0.01, VIP score > 1, and Fc > 1.2 or < 0.83. Of these, 17 DMs were upregulated, and 9 DMs were downregulated. To investigate the relationship between these DMs and key microbial genera, Pearson correlation coefficients were calculated between the DMs and the relative abundance of the top six microbial genera. As shown in Figure 5, *Escherichia* showed a significant correlation with 18 DMs, *Bacillus* with 17, *Pseudomonas* with 12, *Alternaria* with 11, and *Pantoea* with 2. No significant correlations were observed with *Streptomyces*.

Notably, *Bacillus* showed a positive correlation with all 17 upregulated DMs and a negative correlation with all 9 downregulated DMs, reflecting a consistent trend between *Bacillus* and the regulation of these metabolites. Further analysis revealed that *Bacillus* exhibited a significant positive correlation with 13 upregulated DMs and a significant negative correlation with 4 downregulated DMs. Three genera, including *Alternaria*, *Escherichia*, and *Pseudomonas,* were significantly positively correlated with the downregulated metabolites acetylharpagide and lenalidomide. Additionally, these three genera showed significant negative correlations with several upregulated metabolites, including 1-(2,4-difluorobenzoyl)-4-piperidinecarboxylic acid, 5-phenyl-2,4-pentadienoic acid, IAA-Asp, isorhamnetin, isovitexin, kaempferol, LPC 17:0-SN1, sugiol, and trifolin. *Pantoea* was negatively correlated only with euphorbia factor L3 and hesperetin 5-O-glucoside. In summary, this correlation analysis demonstrates a strong relationship between the DMs and the microbial communities.

## 4 Discussion

In recent years, there has been growing interest in improving tobacco leaf quality through biodegradation by microorganisms. Utilizing tobacco-derived microorganisms for tobacco fermentation offers a safer and more adaptable alternative to external inoculants, reducing food safety concerns while enhancing adaptability to the tobacco environment (Ning et al., 2023). In this study, we investigated the *B. velezensis* HJ-16 strain, isolated from tobacco leaves, to examine its extracellular enzyme production, thermal stability, and its impact on sensory properties, metabolite profiles, microbial communities in tobacco leaves, as well as the correlation between these communities and DMs. The results showed that this strain produces various extracellular enzymes, including amylase, cellulase, and protease, with notable enzymatic activity and thermostability. Fermentation with this strain significantly improved the sensory qualities of tobacco leaves, enhancing aromatic intensity, density, softness, and overall quality while reducing irritation (Figures 1A–C).

Scanning electron microscopy (SEM) revealed a looser surface structure in fermented tobacco leaves, with fewer stomata (Figure 1D). This observation was similar to findings by Ning et al., who reported that microbial fermentation of tobacco powder and leaves led to a more loose, wrinkled and porous surface (Ning et al., 2023). This structural loosening is likely due to cellulase breaking down cellulose in the cell walls, altering the physical properties of leaf. Such changes in structure may positively impact tobacco combustion and facilitate the absorption of flavor-enhancing additives.

Genomic analysis of *B. velezensis* HJ-16 confirmed its strong genetic potential for extracellular enzyme production and metabolic activity. Functional annotations through GO, COG, KEGG, and CAZy databases (Figures 2C–F) highlighted the strain’s ability to catalyze reactions and metabolize carbohydrates and proteins, supporting its role in degrading complex molecules in tobacco leaves. Previous studies have highlighted the importance of alpha-amylase (EC 3.2.1.1) in starch degradation during tobacco processing (Ye et al., 2023), consistent with our findings. Similarly, the identification of cellulase-related genes provides insights into the cellulose degradation ability at the genetic level (Ma D. et al., 2024).

The metabolites of tobacco leaves are closely linked to their quality and aroma (Zhang et al., 2018). In this study, metabolomic analysis identified significant changes in tobacco leaf metabolites following *B. velezensis* HJ-16 fermentation (Figure 3). The strain upregulated various organoheterocyclic compounds while downregulating lipids. Notably, heterocyclic compounds such as indoles, pyridines, quinolines, and thiazoles, were directly associated with the aroma of tobacco leaves. KEGG pathway enrichment analysis highlighted the importance of flavonoid and aromatic amino acid metabolism, both critical for flavor enhancement. For example, phenylalanine derivatives contribute significantly to tobacco aroma (Song et al., 2024; Ye et al., 2024). Many flavonoids exhibit strong antioxidant activity due to their chemical structure, which helps to preserve the stability of other compounds in tobacco leaves (Zhang et al., 2022). Alkaloid metabolism pathways were enriched as well, contributing to the complex and intense flavors characteristic of tobacco (Huang et al., 2022). The conversion of macromolecules, like fatty acid conjugates and organic acids in fresh plant leaves, into smaller compounds positively influences their flavor profile (Ye et al., 2024). These metabolic changes, including alterations in lipids, organic acids, and flavonoids, improved the flavor and sensory characteristics of fermented tobacco (Hu et al., 2022). Our findings align with previous studies that reported significant metabolite changes in microbially fermented tobacco (Huang et al., 2022; Song et al., 2024; Wu et al., 2022a).

Changes in tobacco metabolites are closely related to microbial communities. Introducing *B. velezensis* HJ-16 significantly altered the microbial composition on tobacco leaves, which in turn influenced metabolic pathways, leading to the production of differential metabolites (Song et al., 2024). Metagenomic analysis revealed that inoculation with *B. velezensis* HJ-16 significantly altered the microbial community on the tobacco leaf surface (Figure 4). *Bacillus* became the dominant genus, and LEfSe analysis identified *Bacillus* as a key biomarker. Furthermore, correlation analysis between DMs and key microbial genera revealed a significant association between *Bacillus* and specific differential metabolites, underscoring its crucial role in enhancing tobacco leaf quality. KEGG pathway annotations indicated increased microbial metabolic activity, which contributed to the fermentation process and improved leaf quality. Additionally, KEGG annotations revealed a strong overlap between the pathways enriched in microbial functions and those associated with differential metabolites.

In summary, this multi-omics analysis provides valuable insights into the molecular mechanisms by which *B. velezensis* HJ-16 improves tobacco leaf quality. The inoculation of *B. velezensis* HJ-16 significantly altered the microbial community structure, enhancing metabolic pathways, particularly those related to carbohydrate metabolism, amino acid metabolism, and lipid metabolism. These changes, in turn, significantly impacted tobacco leaf metabolites, ultimately improving leaf quality and sensory characteristics.

## Data Availability

Original datasets are available in the following publicly accessible repositories: The whole-genome sequencing data of *Bacillus velezensis* HJ-16 have been submitted to NCBI database (Accession Number: CP169535). The raw sequence reads the microbial communities on tobacco leaves have been deposited in the NCBI Sequence Read Archive (SRA) database (Accession Number: PRJNA1159120). The untargeted metabolomics data have been uploaded to MetaboLights (unique identifier: MTBLS11080) and can be accessed at https://www.ebi.ac.uk/metabolights/MTBLS11080.
