# Supplementary material for: Analysis of the effects of Bacillus velezensis HJ-16 inoculation on tobacco leaves based on multi-omics methods
Source: Front Bioeng Biotechnol. 2024 Dec 6;12:1493766. doi: 10.3389/fbioe.2024.1493766 (PMC11659759; doi:10.3389/fbioe.2024.1493766)
Supplement: Supplementary file 2 [file DataSheet1.docx]

Supplementary Material

## Supplementary Figures


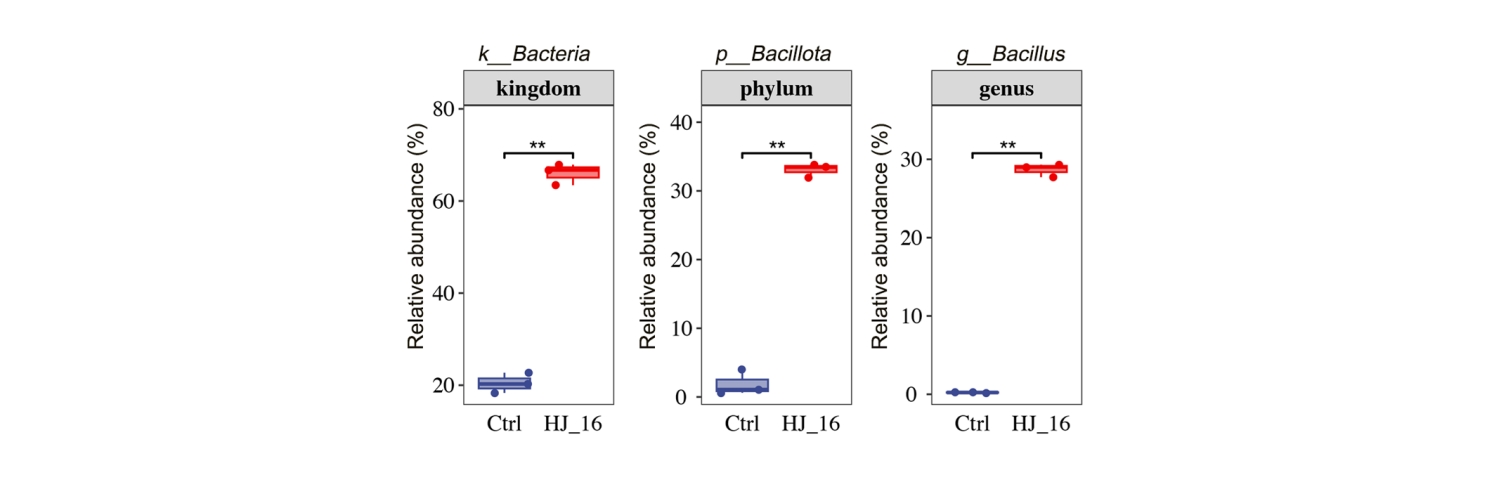


**Supplementary Figure 1.** Species differential analysis. Statistical testing of species abundance data between groups was conducted using the MetaGenomeSeq method, with p-values adjusted to q-values for further analysis. The differential analysis is shown at the kingdom, phylum, and genus levels.
